# Endometrium On-a-Chip Reveals Insulin- and Glucose-induced Alterations in the Transcriptome and Proteomic Secretome

**DOI:** 10.1210/endocr/bqab054

**Published:** 2021-03-10

**Authors:** Tiago H C De Bem, Haidee Tinning, Elton J R Vasconcelos, Dapeng Wang, Niamh Forde

**Affiliations:** 1 Discovery and Translational Sciences Department, Leeds Institute of Cardiovascular and Metabolic Medicine, School of Medicine, University of Leeds, Leeds, UK; 2 Departamento de Medicina Veterinária, Faculdade de Zootecnia e Engenharia de Alimentos, Universidade de São Paulo, Pirassununga, SP, Brazil; 3 LeedsOmics, University of Leeds, Leeds, UK

**Keywords:** Endometrium on-a-chip, microfluidics, uterus, uterine luminal fluid, bovine, cattle

## Abstract

The molecular interactions between the maternal environment and the developing embryo are key for early pregnancy success and are influenced by factors such as maternal metabolic status. Our understanding of the mechanism(s) through which these individual nutritional stressors alter endometrial function and the in utero environment for early pregnancy success is, however, limited. Here we report, for the first time, the use of an endometrium-on-a-chip microfluidics approach to produce a multicellular endometrium in vitro. Isolated endometrial cells (epithelial and stromal) from the uteri of nonpregnant cows in the early luteal phase (Days 4-7) were seeded in the upper chamber of the device (epithelial cells; 4-6 × 10^4^ cells/mL) and stromal cells seeded in the lower chamber (1.5-2 × 10^4^ cells/mL). Exposure of cells to different concentrations of glucose (0.5, 5.0, or 50 mM) or insulin (Vehicle, 1 or 10 ng/mL) was performed at a flow rate of 1 µL/minute for 72 hours. Quantitative differences in the cellular transcriptome and the secreted proteome of in vitro*–*derived uterine luminal fluid were determined by RNA-sequencing and tandem mass tagging mass spectrometry, respectively. High glucose concentrations altered 21 and 191 protein-coding genes in epithelial and stromal cells, respectively (*P* < .05), with a dose-dependent quantitative change in the protein secretome (1 and 23 proteins). Altering insulin concentrations resulted in limited transcriptional changes including transcripts for insulin-like binding proteins that were cell specific but altered the quantitative secretion of 196 proteins. These findings highlight 1 potential mechanism by which changes to maternal glucose and insulin alter uterine function.

Successful establishment of pregnancy in placental mammals requires bilateral interactions between the developing embryo and the maternal endometrium. While direct contact with the maternal environment is not strictly required, ie, viable embryos can be successfully produced in vitro, interactions with the maternal tract substantially enhances the quality of the embryo (1-3). This increased developmental competency is mediated, in part, via the transport and secretion of endometrial derived molecules (including proteins, amino acids, metabolites, lipids, and RNA species encapsulated in extracellular vesicles) that are taken up by the embryo and support development prior to establishment of the placenta (4, 5). The composition of uterine luminal fluid (ULF) and the molecular interactions between the mother and developing embryo are known to be influenced by maternal factors such as the metabolic status of the mother and the quality of the embryo present (reviewed by (6, 7)). Exposure to adverse conditions, such as nutritional insults, at specific developmental time points can alter an individual’s susceptibility to disease in later life (7). Despite a significant volume of literature describing the composition of ULF (8, 9), efforts to supplement culture media with known components have not substantially improved development, suggesting there are likely still unknown components of ULF yet to be discovered.

The uterine epithelium, at least for a few key days, is potentially the most critical maternal tissue in the establishment of a healthy pregnancy (10). Thus, exposure of the endometrium to stressors can alter the developmental or epigenetic programming of the fetus. In the dairy cow, the early postpartum period is frequently associated with nutrition-associated metabolic stress as cows cannot take in sufficient dietary energy to offset the demands of peak milk production. This induces a maternal metabolic environment characterized by high nonesterified fatty acids (NEFAs), beta-hydroxybutyrate, and low insulin, insulin-like growth factor (IGF)-I, and glucose (11, 12). Given the livestock production cycle, this postpartum altered environment typically occurs at the same time as which the next pregnancy is being established. There is a growing body of evidence to suggest that this metabolic stress compromises the ability of the reproductive tract of the lactating dairy cow to support early development (11, 12) associated with alterations in global gene expression in the embryo/conceptus, the oviduct (9, 13), and endometrium (13-15). Conceptuses derived from early in lactation are less developmentally competent (metabolic stress) than late stages of lactation (16). Even if a high-quality embryo is produced, exposure to a suboptimal uterine environment such as that in high-producing lactating cows can compromise developmental potential (16, 17).

Our understanding of the mechanism(s) through which individual metabolites alter endometrial function and the in utero environment is relatively limited. While traditional static cell culture models have been used to address the issue of conceptus–maternal interaction, they do have limitations; for example, they do not mimic the dynamic nature of these metabolic components to which the endometrium is exposed. Nor do they allow for assessment of how these metabolic extremes alter the interactions between the heterogeneous cells types of the endometrium and the ULF that is produced as a consequence. Advances in microfluidics and organ-on-a-chip technologies in reproductive systems have facilitated the study of embryo development as well as cervical (18), ovarian (19), endometrial (20), and placental function (21, 22) in humans and mice. Such systems have been used to mimic the bovine oviduct environment (23) and the menstrual cycle in vitro using a combination of human (fallopian tube, endometrium, ectocervix, liver) and murine (ovary) components (24). However, the power of these systems has not yet been exploited to investigate how maternal nutritional stressors alter the uterine environment to which the embryo is exposed, either at the level of the transcriptomic or proteomic secretome.

Here, we report for the first time the use of microfluidics to produce a multicellular endometrium in vitro that was exposed to glucose and insulin concentrations associated with maternal metabolic stressors. We specifically have focused on recapitulating Days 4-7 of pregnancy when the embryo enters the uterus in vivo, transitions between the morula and blastocyst stages, and is susceptible to reprogramming events (6). We used RNA sequencing to determine how these metabolites alter the cell-specific transcriptional response in the endometrium to these nutritional stressors. We further demonstrate how these changes alter the proteomic content of in vitro*–*derived ULF secreted from the endometrial epithelium. Collectively, these data highlight 1 potential mechanism by which changes to maternal glucose and insulin concentrations alter uterine function. We propose that these are candidate proteins that can modify the developmental potential of embryos.

## Materials and Methods

Unless otherwise stated, all chemical and reagents were purchased from Sigma-Aldrich Chemical Co. (St. Louis, MO). The in vitro experimental procedures were conducted in humidified incubators maintained at 38.5°C with 5% CO_2_ in air.

### Primary Endometrial Cell Isolation and Culture

**Figure 1.**
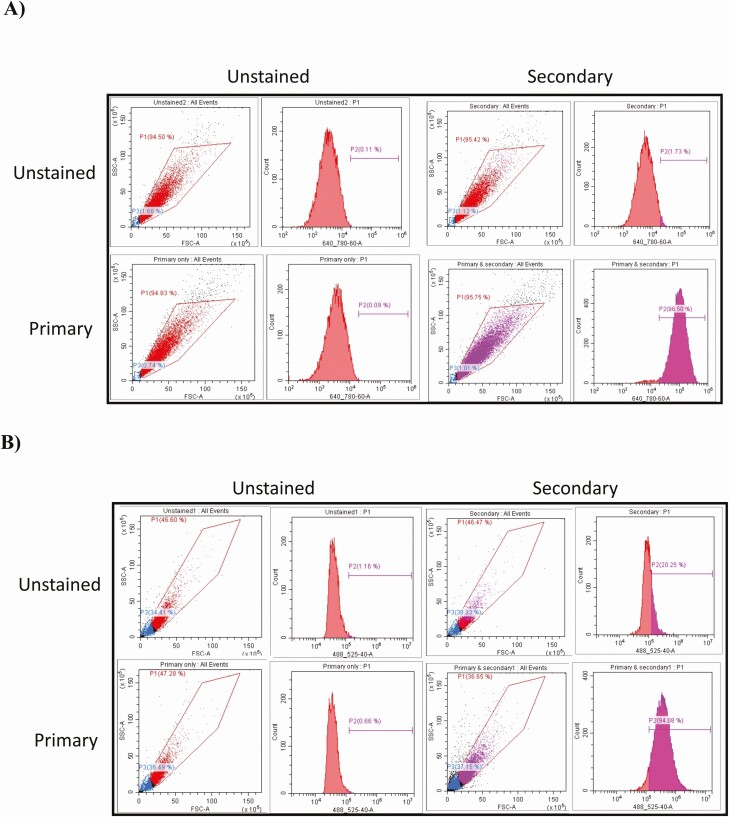
Flow cytometry validation of bovine endometrial (A) stromal enriched or (B) epithelial enriched cell types. Primary bovine endometrial cells were isolated as described and cultured to confluency. Cells were lifted using trypsin (0.025%) in PBS, washed in PBS, and 1 × 10^6^ cells from each cell type fixed and permeabilized using the FIX & PERM kit as per the manufacturers protocol (ThermoFisher Scientific). (A) Antikeratin 18 rabbit IgG (1 μg/mL) (SAB5500126) was added to the epithelial-enriched cell fraction, incubated for 15 minutes, washed with PBS, and incubated with anti-rabbit IgG with 680 nm flurophore (1 μg/mL) (SAB4600395) for 15 minutes. After a final wash cells were immediately analyzed on a CytoFLEX S (Beckman Coulter) (640-nm laser). (B) Antivimentin mouse IgG (1 μg/mL) (SAB4200761) was added to the stromal enriched cell fraction, incubated for 15 minutes, washed in PBS, and incubated with antimouse IgG with 488 nm flurophore (1 μg/mL) (SAB4600029) for 15 minutes. After a final wash cells were immediately analyzed on a CytoFLEX S (488 nm laser). Appropriate gating was used to exclude cell debris and clumps of cells, and control samples including no antibody, primary only, and secondary only were included. The % of positively stained cells were determined by gating the area to the right of the unstained peak.

Endometrial cell isolation was carried out as previously described (25). Briefly, uteri from nonpregnant cows (*Bos taurus*), early in the luteal phase (Days 4-7 approximately) were selected on the basis of corpus luteum morphology as previously described (26). This stage of the cycle was chosen as it is when the embryo is present in the uterus and undergoes the transition from morula to blastocyst, a key developmental timepoint where is can be susceptible to extremes in the maternal environment. Endometrial tissue was dissected from the underlying myometrium and incubated in 25 mL of digest solution containing bovine serum albumin (1 mg/mL), trypsin EDTA (2.5 BAEE units/mL), collagenase (0.5 mg/mL) and DNase I (0.1 mg/mL) in Hanks’ buffered saline solution in a shaking water bath for 1 hour at 37°C. The digestion solution was filtered through a 100-μm-mesh cell strainer over a 4-μm cell strainer, washed twice with Hanks’ buffered saline solution (containing 10% fetal bovine serum), and centrifuged at 700*g* for 7 minutes. The resulting cell pellet containing stromal cells was resuspended in RPMI 1640 culture medium containing 10% fetal bovine serum, streptomycin (50 μg/mL), penicillin (50 IU/mL), and amphotericin B (2.5 μg/mL). The 40-μm strainer was inverted and flushed with culture medium to recover epithelial cells. The cell populations were seeded at 1 × 10^5^ cells/mL into 75-cm^2^ culture flasks (Greiner BioOne, Gloucestershire, UK). After 5-7 days of culture, cell populations were further purified using their differential plating times. To characterized the cellular populations, cells were washed in phosphate-buffered saline (PBS), and 1 × 10^6^ cells from each cell type fixed and permeabilized using the FIX & PERM kit as per the manufacturers protocol (ThermoFisher Scientific). Primary antibodies were added to the cells following permeabilization at the concentrations recommended by supplier, incubated for 15 minutes, washed, secondary antibody added, and incubated for the following 15 minutes. Cells were then immediately analyzed on a CytoFLEX S (Beckman Coulter) with appropriate gating to remove clumps of cells and cellular debris. To the epithelial cells specifically, the cells were stained with antikeratin 18 rabbit immunoglobulin (Ig)G primary antibody and antirabbit IgG secondary antibody. The 640-nm laser was used to analyze the samples. The stromal cells were stained with antivimentin primary IgG and antimouse IgG secondary antibody. The 488-nm laser was used on the stromal cell samples (Fig. 1).

### Cell Seeding into the Microfluidic Device

**Figure 2.**
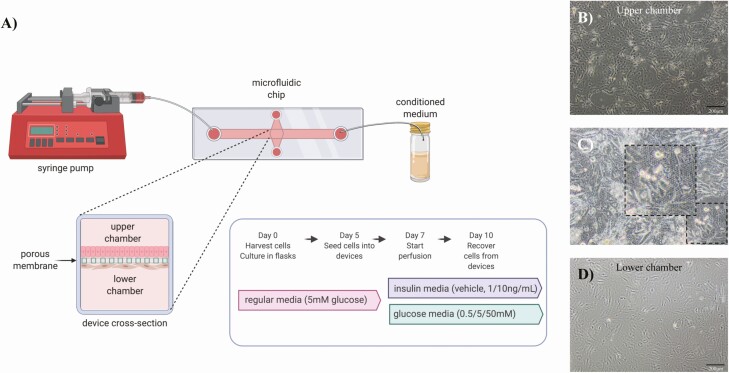
(A) Schematic diagram of endometrium-on-a-chip microfluidic device and experimental design used to mimic the physiological extremes of glucose and insulin. Representative images of (B) epithelial cells seeded into the upper chamber, (C) cells attached on and under the porous membrane, and (D) stromal cells seeded into the lower chamber. The rate of flow for both experiments was performed at 1 μL/min per 72 hours.

All cells were seeded into the devices in RPMI 1640 medium as described above (Fig. 2). Stromal enriched cells were seeded in the lower chamber of the device (10-µm slide membrane, IBIDI), using a 1-mL syringe, at concentration of 1.5-2 × 10^4^ cells/mL in a final volume of 300 µL. All devices were inverted for 2 hours to allow stromal cells to adhere to the underside of the porous membrane. Devices were placed in the normal orientation and epithelial cells were seeded at 4-6 × 10^4^ cells/mL in a final volume of 55 µL into the upper chamber. Cells were left to become 60% confluent over 2 days with 1 medium change (48 hours) before beginning the flow perfusion. For the glucose experiment, on the day of experimentation medium was changed and 5 mL of medium without glucose was loaded into 5-mL syringes at the inlet (n = 2 technical replicates) with either 0.5, 5.0, or 50.0 mM of glucose. These were chosen as a dose response of glucose with 5.0 mM reflecting normal concentrations of glucose detected in the ULF (3.79 mM) and plasma (6.34 mM) during the early luteal phase of the cycle (27). For the insulin experiment, on the day of experimentation medium was also changed and 5 mL of medium with a physiologic concentration of glucose (5.0 mM) was loaded into 5-mL syringes at the inlet (n = 2 technical replicates) with either a vehicle control (acetic acid in PBS pH 3.0), 1.0 ng/mL, or 10.0 ng/mL insulin. These were chosen as a dose response representing extremes of insulin observed in circulation of cows undergoing negative energy balance (28). The pump was set to flow medium through the device at a flow rate of 1 µL/minute to mimic the rate of secretion in vivo (29) for 72 hours. Culture medium from the upper chamber (in vitro*–*derived ULF) was recovered with a pipette and snap-frozen in liquid nitrogen and the samples were stored at –80°C. Epithelial and stromal cells were separately removed from the device via 0.5% trypsin, centrifuged at 2000*g* for 10 minutes, snap-frozen in liquid nitrogen, and stored at –80°C prior to processing.

### RNA Extraction and Sequencing

Total RNA was extracted from epithelial and stromal cells using the Mini RNeasy kit (Qiagen, Crawley, UK) following the manufacturer’s recommendations. Cell samples were homogenized in 700 µL of Qiazol via vortexing for 1 minute at room temperature (RT). On-column DNase digestion was performed (15 minutes at RT) and RNA was eluted in 14 µL of RNase/DNase free water from the spin column membrane following centrifugation for 2 minutes at full speed and this step was performed twice.

RNA sequencing was performed as previously described (25) with minor modifications. Briefly, RNA quality and quantity were confirmed using the Agilent Bioanalyzer system, and all samples had an RNA integrity number of >7.9. Stranded RNA sequencing libraries were constructed using the Encore Complete RNA-Seq library system of NuGEN. All libraries were sequenced on NextSeq generating 75 bp single-end reads. The raw FASTQ files were inspected using FastQC (https://www.bioinformatics.babraham.ac.uk/projects/fastqc/), the adapter sequences were trimmed using Cutadapt (30), and additional quality control steps taken by fastq_quality_filter program as part of FASTX-Toolkit (http://hannonlab.cshl.edu/fastx_toolkit/). The mapping process was performed using align function in Rsubread (31) package by aligning the clean fastq files against the cow reference genome retrieved from Ensembl release 96 (32) (*Bos taurus*) and only uniquely mapped alignments were recorded. The resulting BAM files were sorted and indexed by SAMtools (33). The reads were summarized at the gene level by means of featureCounts (34). DESeq2 (35) in a paired sample design was used to identify differentially expressed protein-coding genes based on the cut-offs of a *P* < .05 and a log_2_ fold change >0.1 or <–0.1. Variance stabilizing transformations were applied to the genes that had at least 10 reads in total for all samples. Heatmaps were created using the transformed read counts based on a pool of differentially expressed protein-coding genes for each experiment. Principal component analysis (PCA) analysis was carried out for the protein-coding genes that have Reads Per Kilobase Million (RPKM) ≥ 1 in at least 1 sample and 2 normalization approaches such as log2(RPKM+1) and quantile normalization were conducted prior to PCA analysis.

### Quantitative Proteomic Analysis of In Vitro–derived Uterine Luminal Fluid Recovered from the Upper Chamber

Medium (n = 3 samples per group) from the upper chamber (in vitro*–*derived ULF) following glucose or insulin exposure were subjected to albumin depletion according to the manufacturer’s instructions (Thermo Fisher Scientific, Loughborough, UK). Individual samples were digested with trypsin (2.5 µg of trypsin; 37°C, overnight), labelled with tandem mass tag (TMT) 10-plex reagents according to the manufacturer’s protocol (Thermo Fisher Scientific) and pooled. The pooled sample was evaporated to dryness, resuspended in 5% formic acid, and then desalted using a SepPak cartridge according to the manufacturer’s instructions (Waters, Milford, MA, USA). Eluate from the SepPak cartridge was again evaporated to dryness and resuspended in buffer A (20 mM ammonium hydroxide, pH 10) prior to fractionation by high pH reversed-phase chromatography using an Ultimate 3000 liquid chromatography system (Thermo Scientific). In brief, the sample was loaded onto an XBridge BEH C18 Column (130 Å, 3.5 µm, 2.1 mm × 150 mm, Waters, UK) in buffer A and peptides eluted with an increasing gradient of buffer B (20 mM ammonium hydroxide in acetonitrile, pH 10) from 0 to 95% over 60 minutes. The resulting fractions were evaporated to dryness and resuspended in 1% formic acid prior to analysis by nano-liquid chromatography (LC) tandem mass spectrometry (MS/MS) using an Orbitrap Fusion Lumos mass spectrometer (Thermo Fisher Scientific).

### Nano-liquid Chromatography Mass Spectrometry

High pH reversed phase fractions were further fractionated using an Ultimate 3000 nano-LC system in line with an Orbitrap Fusion Lumos mass spectrometer (Thermo Scientific). Peptides in 1% (vol/vol) formic acid were injected onto an Acclaim PepMap C18 nano-trap column (Thermo Scientific). After washing with 0.5% (vol/vol) acetonitrile 0.1% (vol/vol), formic acid peptides were resolved on a 250 mm × 75 μm Acclaim PepMap C18 reversed phase analytical column (Thermo Scientific) over a 150 minute organic gradient, using 7 gradient segments (1-6% solvent B over 1 minute, 6-15% B over 58 minutes, 15-32% B over 58 minutes, 32-40% B over 5 minutes, 40-90% B over 1 minute, held at 90% B for 6 minutes and then reduced to 1% B over 1 minute) with a flow rate of 300 nL/minute. Solvent A was 0.1% formic acid and solvent B was aqueous 80% acetonitrile in 0.1% formic acid. Peptides were ionized by nano-electrospray ionization at 2.0 kV using a stainless-steel emitter with an internal diameter of 30 μm (Thermo Scientific) and a capillary temperature of 275°C. All spectra were acquired using an Orbitrap Fusion Lumos mass spectrometer controlled by Xcalibur 4.1 software (Thermo Scientific) and operated in data-dependent acquisition mode using an SPS-MS3 workflow. Fourier transform mass analyzer (FTMS) 1 spectra were collected at a resolution of 120 000, with an automatic gain control (AGC) target of 200 000 and a max injection time of 50 ms. Precursors were filtered with an intensity threshold of 5000, according to charge state (to include charge states 2-7) and with monoisotopic peak determination set to peptide. Previously interrogated precursors were excluded using a dynamic window (60 seconds ± 10 ppm). The MS2 precursors were isolated with a quadrupole isolation window of 0.7 m/z. ITMS2 spectra were collected with an AGC target of 10 000, maximum injection time of 70 ms and collision-induced dissociation (CID) collision energy of 35%. For FTMS3 analysis, the Orbitrap was operated at 50 000 resolution with an AGC target of 50 000 and a maximum injection time of 105 ms. Precursors were fragmented by high-energy collision dissociation at a normalized collision energy of 60% to ensure maximal TMT reporter ion yield. Synchronous precursor selection was enabled to include up to 5 MS2 fragment ions in the FTMS3 scan.

The raw data files were processed and quantified using Proteome Discoverer software v2.1 (Thermo Scientific) and searched against the UniProt *Bos taurus* database (downloaded June 2019: 46 309 entries) using the SEQUEST algorithm. Peptide precursor mass tolerance was set at 10 ppm, and MS/MS tolerance was set at 0.6 Da. Search criteria included oxidation of methionine (+15.9949) as a variable modification and carbamidomethylation of cysteine (+57.0214) and the addition of the TMT mass tag (+229.163) to peptide N-termini and lysine as fixed modifications. Searches were performed with full tryptic digestion and a maximum of 2 missed cleavages were allowed. The reverse database search option was enabled, and all data was filtered to satisfy false discovery rate (FDR) of 5%. Differences in protein abundance among groups were determined using an unpaired T-test following the FDR filtration step. The mass spectrometry proteomics data have been deposited to the ProteomeXchange Consortium via the PRIDE partner repository with the dataset identifier PXD024218.

### Regulatory Networks and Functional Annotations of Biological Processes Analysis Were Performed by Bioinformatics Using DAVID

Gene enrichment analysis was performed using DAVID (Database for annotation, visualization, and integrated discovery; Bioinformatics Resources 6.7) (36) to predict the regulatory networks (signaling pathways) and specific functional annotations from gene ontology (GO) terms related to the proteins and differentially expressed genes (DEGs), respectively. The accession number from each protein (Uniprot) or gene (Ensembl Gene ID) were used. All the signaling pathways GO terms identified from biological process, cellular component, and molecular function were considered enriched a *P* < .05 threshold. To perform and visualization of the networks of the signaling pathways was used the software platform Cytoscape version 3.7.2 (37). The signaling pathways were presented by the Fold Enrichment each networking and the GO terms were arranged by the Enrichment Score (–log_10_^[*P*value]^), respectively.

## Results

### Characterization of Cellular Phenotypes in the Microfluidics Devices

Fluorescence-activated cell sorting analysis demonstrated that the epithelial cell population stained 96.5% positive for Keratin 18 with <2.0% secondary antibody positivity. The stromal enriched cell population were 94% positive for vimentin with 20% secondary antibody positivity (Fig. 1). The epithelial and stromal cells both expressed mRNA for the progesterone receptor (*PGR*) and estrogen receptor (*ESR1*); however, there was no difference in expression of these between treatments (all normalized data are available via GEO accession number GSE167086).

### Exposure of Endometrial Epithelial and Stromal Cells In Vitro to High Concentrations of Glucose Alters the Transcriptional Profile in a Cell-specific Manner

**Figure 3.**
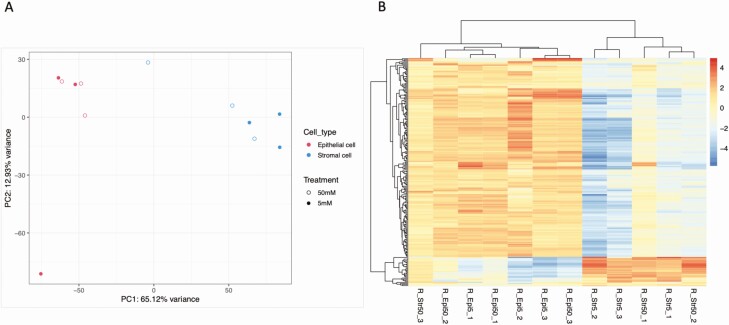
(A) Principal component analysis (PCA) plot of overall transcriptional profile determined via RNA sequencing of bovine endometrial epithelial and stromal cells exposed to either 5 mM or 50 mM of glucose for 72 hours under flow (n = 3 biological replicates). Epithelial (left hand side) and stromal cells (right hand side) clustered into 2 distinct populations. (B) Heatmap showing the transcript expression for individual samples with lower levels (blue) and those with higher expression shown in red. Samples from epithelial cells (n = 6) and samples from stromal cells (n = 6) are arranged from left to the right.

**Figure 4.**
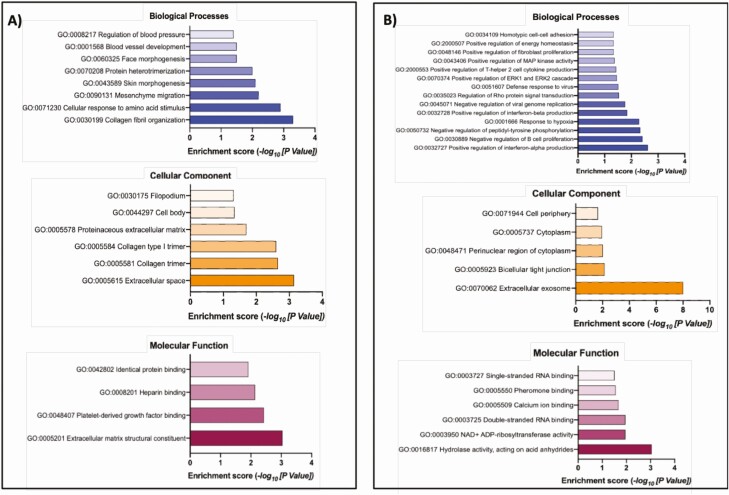
Gene ontology overrepresented Biological Process, Cellular Component, and Molecular Function from DEGs in (A) epithelial (n = 21) and (B) stromal cells (n = 191) following exposure to different concentrations of glucose in vitro. The transcript accession numbers (Ensembl Transcript ID) were inputted into DAVID—Functional Annotation Tool (DAVID Bioinformatics Resources 6.7, NIAID/NIH—https://david.ncifcrf.gov/summary.jsp) and those that were significantly overrepresented in the list of DEGs are presented above for biological processes (blue bars), cellular component (orange bars) and molecular function (plum bars) with associated enrichment score.

PCA (Fig. 3) revealed distinct clustering of epithelial and stromal cells but there was no clear clustering between the glucose treatments (5 mM vs 50 mM). Flow of high concentrations of glucose (50 mM) for 3 days altered the expression of 21 and 191 protein-coding genes in the epithelial and stromal cells, respectively, compared to control concentrations (5.0 mM glucose) (available at GEO database under accession number GSE167086). Functional annotation analysis determined n = 8 and n = 14 overrepresented biological processes, n = 6 and n = 5 overrepresented cellular components and n = 4 and n = 6 overrepresented molecular functions, for epithelial and stromal cells exposed to 50 mM glucose, respectively (Fig. 4; Table 1).

**Table 1.** Functional annotation of GO terms related to the differentially expressed genes following exposure on endometrium on-a-chip to high concentrations of glucose (5 mM vs 50 mM of glucose)

Category	Term	Count	%	*P* value
**Epithelial cells**				
GOTERM_BP_DIRECT	GO:0030199~collagen fibril organization	3	15.0	.000500
GOTERM_BP_DIRECT	GO:0071230~cellular response to amino acid stimulus	3	15.0	.001200
GOTERM_BP_DIRECT	GO:0090131~mesenchyme migration	2	10.0	.005800
GOTERM_BP_DIRECT	GO:0043589~skin morphogenesis	2	10.0	.008100
GOTERM_BP_DIRECT	GO:0070208~protein heterotrimerization	2	10.0	.010000
GOTERM_BP_DIRECT	GO:0060325~face morphogenesis	2	10.0	.032000
GOTERM_BP_DIRECT	GO:0001568~blood vessel development	2	10.0	.032000
GOTERM_BP_DIRECT	GO:0008217~regulation of blood pressure	2	10.0	.039000
GOTERM_BP_DIRECT	GO:0090263~positive regulation of canonical Wnt signaling pathway	2	10.0	.065000
GOTERM_CC_DIRECT	GO:0005615~extracellular space	7	35.0	.000720
GOTERM_CC_DIRECT	GO:0005581~collagen trimer	3	15.0	.002200
GOTERM_CC_DIRECT	GO:0005584~collagen type I trimer	2	10.0	.002500
GOTERM_CC_DIRECT	GO:0005578~proteinaceous extracellular matrix	3	15.0	.020000
GOTERM_CC_DIRECT	GO:0044297~cell body	2	10.0	.046000
GOTERM_CC_DIRECT	GO:0030175~filopodium	2	10.0	.049000
GOTERM_CC_DIRECT	GO:0030018~Z disc	2	10.0	.088000
GOTERM_MF_DIRECT	GO:0005201~extracellular matrix structural constituent	3	15.0	.000910
GOTERM_MF_DIRECT	GO:0048407~platelet-derived growth factor binding	2	10.0	.003700
GOTERM_MF_DIRECT	GO:0008201~heparin binding	3	15.0	.007200
GOTERM_MF_DIRECT	GO:0042802~identical protein binding	3	15.0	.012000
**Stromal cells**				
GOTERM_BP_DIRECT	GO:0032727~positive regulation of interferon-alpha production	3	1.7	.00240
GOTERM_BP_DIRECT	GO:0030889~negative regulation of B cell proliferation	3	1.7	.00380
GOTERM_BP_DIRECT	GO:0050732~negative regulation of peptidyl-tyrosine phosphorylation	3	1.7	.00460
GOTERM_BP_DIRECT	GO:0001666~response to hypoxia	5	2.9	.00510
GOTERM_BP_DIRECT	GO:0032728~positive regulation of interferon-beta production	3	1.7	.01400
GOTERM_BP_DIRECT	GO:0045071~negative regulation of viral genome replication	3	1.7	.01700
GOTERM_BP_DIRECT	GO:0035023~regulation of Rho protein signal transduction	4	2.3	.02900
GOTERM_BP_DIRECT	GO:0051607~defense response to virus	5	2.9	.03100
GOTERM_BP_DIRECT	GO:0070374~positive regulation of ERK1 and ERK2 cascade	5	2.9	.03500
GOTERM_BP_DIRECT	GO:2000553~positive regulation of T-helper 2 cell cytokine production	2	1.2	.03700
GOTERM_BP_DIRECT	GO:0043406~positive regulation of MAP kinase activity	3	1.7	.04300
GOTERM_BP_DIRECT	GO:0048146~positive regulation of fibroblast proliferation	3	1.7	.04600
GOTERM_BP_DIRECT	GO:2000507~positive regulation of energy homeostasis	2	1.2	.04600
GOTERM_BP_DIRECT	GO:0034109~homotypic cell-cell adhesion	2	1.2	.04600
GOTERM_BP_DIRECT	GO:0042127~regulation of cell proliferation	5	2.9	.05000
GOTERM_BP_DIRECT	GO:0034340~response to type I interferon	2	1.2	.05600
GOTERM_BP_DIRECT	GO:0050891~multicellular organismal water homeostasis	2	1.2	.05600
GOTERM_BP_DIRECT	GO:0045087~innate immune response	6	3.5	.06500
GOTERM_BP_DIRECT	GO:0001755~neural crest cell migration	3	1.7	.06500
GOTERM_BP_DIRECT	GO:0009615~response to virus	3	1.7	.06500
GOTERM_BP_DIRECT	GO:0051639~actin filament network formation	2	1.2	.07300
GOTERM_BP_DIRECT	GO:0002376~immune system process	2	1.2	.08200
GOTERM_BP_DIRECT	GO:0016064~immunoglobulin mediated immune response	2	1.2	.08200
GOTERM_BP_DIRECT	GO:0048545~response to steroid hormone	2	1.2	.08200
GOTERM_BP_DIRECT	GO:0051764~actin crosslink formation	2	1.2	.09100
GOTERM_BP_DIRECT	GO:0060158~phospholipase C-activating dopamine receptor signaling pathway	2	1.2	.09100
GOTERM_CC_DIRECT	GO:0070062~extracellular exosome	49	28.5	.000000
GOTERM_CC_DIRECT	GO:0005923~bicellular tight junction	5	2.9	.007100
GOTERM_CC_DIRECT	GO:0048471~perinuclear region of cytoplasm	10	5.8	.009500
GOTERM_CC_DIRECT	GO:0005737~cytoplasm	43	25.0	.011000
GOTERM_CC_DIRECT	GO:0071944~cell periphery	3	1.7	.022000
GOTERM_CC_DIRECT	GO:0030175~filopodium	3	1.7	.059000
GOTERM_CC_DIRECT	GO:0005886~plasma membrane	30	17.4	.063000
GOTERM_CC_DIRECT	GO:0016324~apical plasma membrane	5	2.9	.073000
GOTERM_CC_DIRECT	GO:0031528~microvillus membrane	2	1.2	.083000
GOTERM_CC_DIRECT	GO:0045121~membrane raft	4	2.3	.090000
GOTERM_MF_DIRECT	GO:0016817~hydrolase activity, acting on acid anhydrides	3	1.7	.000890
GOTERM_MF_DIRECT	GO:0003950~NAD + ADP-ribosyltransferase activity	3	1.7	.011000
GOTERM_MF_DIRECT	GO:0003725~double-stranded RNA binding	4	2.3	.011000
GOTERM_MF_DIRECT	GO:0005509~calcium ion binding	12	7.0	.021000
GOTERM_MF_DIRECT	GO:0005550~pheromone binding	2	1.2	.028000
GOTERM_MF_DIRECT	GO:0003727~single-stranded RNA binding	3	1.7	.031000
GOTERM_MF_DIRECT	GO:0030246~carbohydrate binding	4	2.3	.058000
GOTERM_MF_DIRECT	GO:0036094~small molecule binding	2	1.2	.083000
GOTERM_MF_DIRECT	GO:0003924~GTPase activity	5	2.9	.085000

Abbreviations: BP, biological process; CCcellular component; GO, gene ontology; MF, molecular function.

### Exposure to High Concentrations of Glucose Alters the Proteomic Content of In Vitro–derived ULF

**Figure 5.**
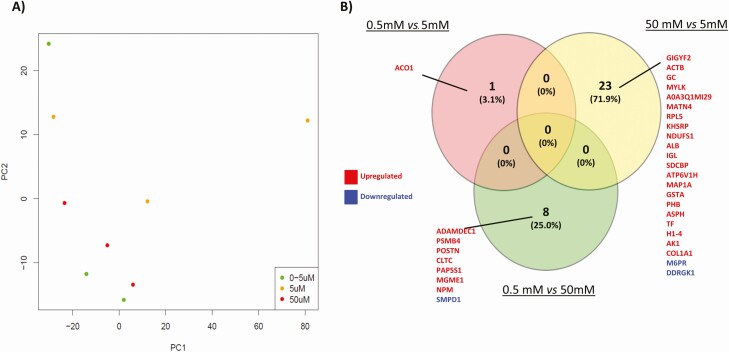
(A) Principal component analysis (PCA) plot shows the distribution of in vitro-derived ULF following tandem mass tag (TMT) mass spectrometry analysis of the proteomic content following exposure to: 0.5 mM (green circle), 5.0 mM (yellow circle), or 50 mM (red circle) concentrations of glucose for 72 hour (n = 3 biological replicates). (B) Venn diagram analysis of overlap and unique proteins that are differentially abundance following exposure to different concentrations of glucose. Fold change differences in protein abundances between groups was determined using paired t-tests and were considered significant when *P* < .05.

PCA (Fig. 5A) revealed that exposure of endometrium on-a-chip to physiological extremes of glucose (0.5 mM and 50 mM) altered the overall composition of proteins in the in vitro*–*derived ULF. The highest concentration of glucose (50 mM) changed the abundance of 23 proteins compared with controls (5 mM), the majority of which were increased (*P* < .05: Fig. 5B). When the lower concentration (0.5 mM) was compared with the control (5 mM), only 1 protein was altered. Finally, when the physiologic extremes (0.5 mM vs 50 mM) were compared, 8 proteins were found to be differentially abundant in in vitro*–*derived ULF. Functional annotation analysis revealed the proteins upregulated following glucose treatment were involved in platelet and lysosome pathways (Table 2). There were no overrepresented pathways associated with the proteins that were decreased in abundance following glucose exposure (*P* > .05).

**Table 2.** Functional annotation analysis of the proteins upregulated in the in vitro uterine luminal fluid produced following glucose treatment

Category	Term	Genes	Count	%	*P* value	Fold enrichment	Benjamini	FDR
**50 mM vs 5 mM**								
KEGG_PATHWAY	bta04611:Platelet activation	Q28824, Q95ND9	2	22.2	.0500	29.7	0.64	32
KEGG_PATHWAY	bta04510:Focal adhesion	Q28824, Q95ND9	2	22.2	.0800	18.1	0.57	47
**0.5 mM vs 50 mM**								
KEGG_PATHWAY	bta04142: Lysosome	P49951, Q0VD19	2	66.7	.03300	39.9	0.29000	18

Proteins were over-represented in pathways associated with platelet activation and focal adhesion (5 mM vs 50 mM of glucose) and the lysosome pathways (0.5 mM vs 50 mM).

### Exposure of the Endometrium to Different Concentrations of Insulin Alters the Transcriptome in a Cell-specific Manner

**Figure 6.**
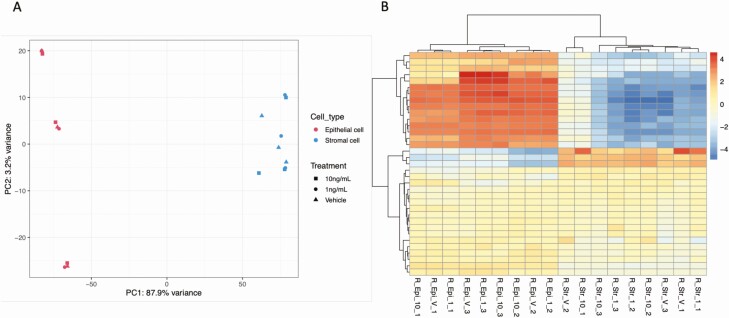
(A) Principal component analysis (PCA) plot of overall transcriptional profile determined via RNA sequencing of bovine endometrial epithelial and stromal cells exposed to either vehicle control, 1 ng/mL, or 10 ng/mL of Insulin for 72 hour under flow (n = 3 biological replicates). Epithelial (left hand side) and stromal cells (right hand side) clustered into 2 distinct populations. (B) Heatmap showing the transcript expression for individual samples with lower levels (blue) and those with higher expression shown in red. Samples from epithelial cells (n = 9) and samples from stromal cells (n = 9) are arranged from left to the right.

The PCA plot showed clear separation of epithelial cells (on the left-hand side of the plot) and stromal cell populations (on the right-hand side of the plot) with limited treatment effect (Fig. 6A). Exposure of cells to 1 ng/mL of insulin for 72 hours changed expression of 4 transcripts (nonspecific serine/threonine protein kinase [*ARAF*], Ubiquitin-40S ribosomal protein S27a [*RPS27A*], NADH-ubiquinone oxidoreductase chain 4L [*M-ND4L*], and NADH-ubiquinone oxidoreductase chain 6 [*MTND6*]) in stromal cells, and 1 unknown transcript (ENSBTAG00000052092) in epithelial cells compared to vehicle control. Exposure to the higher concentrations of insulin (10 ng/mL) altered 10 transcripts in epithelial cells (NADH-ubiquinone oxidoreductase chain 2 [*MT-ND2*]), NADH-ubiquinone oxidoreductase chain 3 [*MT-ND3*], NADH-ubiquinone oxidoreductase chain 5 [*ND5*], CCAAT/enhancer-binding protein beta [*CEBPB*], Ferritin heavy chain 1 [*FTH1*], ATP synthase protein 8 [*MT-ATP8*], Nuclear receptor subfamily 4 group A member 2 [*NR4A2*], Fibronectin [*FN1*], Collagenase 3 [*MMP13*], and Cytochrome b [*MT-CYB*]) and 2 transcripts in stromal cells: insulin-like growth factor-binding protein 6 (*IGFBP6*), and receptor for retinol uptake (*STRA6*).

In addition, when the physiological extremes of insulin (1 ng vs 10 ng/mL) were compared, 1 transcript was differentially expressed in epithelial cells (insulin-like growth factor-binding protein 3 [*IGFBP3*]) while 19 transcripts were altered in the stromal cells (insulin-like growth factor-binding protein 5 [*IGFBP5*], tensin 4 [*TNS4*], ETS domain-containing transcription factor [*EHF*], cytochrome P450, subfamily IIIA, polypeptide 4 [*CYP3A4*], sphingolipid delta (4)-desaturase [*DES1*], CCAAT/enhancer-binding protein beta, C/EBP beta [*CEBPB*], creatine kinase U-type, mitochondrial [*CKMT1A*], claudin-7 [*CLDN7*], mucin 16 [*MUC16*], glycine amidinotransferase, mitochondrial [*GATM*], claudin 6 [*CLDN6*], receptor protein-tyrosine kinase [*ERBB3*], ATP binding cassette subfamily A member 1 [*ABCA1*], epithelial membrane protein 1 [*EMP1*], solute carrier family 2, facilitated glucose transporter member 3 [*SLC2A3*], adseverin [*SCIN*], aldehyde dehydrogenase 1 family member A3 [*ALDH1A3*], vascular endothelial growth factor A [*VEGFA*], and NADH-ubiquinone oxidoreductase chain 6 [*MT-ND6*]). Functional annotation analysis found no overrepresented terms associated with the lower concentration of insulin than control. However, DEGs associated with the higher concentration of insulin were overrepresented in 3 cellular components (Respiratory chain, Mitochondrial respiratory chain complex I, and Mitochondrial inner membrane) and 1 molecular function (NADH dehydrogenase [ubiquinone] activity).

### Altering Insulin Concentrations Modifies the Secretome of In Vitro–derived ULF

**Figure 7.**
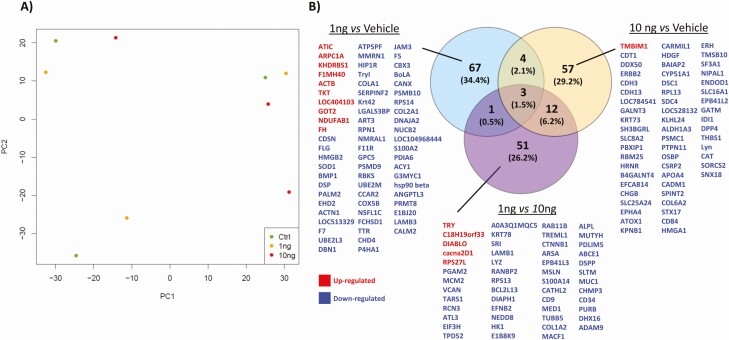
(A) Principal component analysis (PCA) plot shows the distribution of in vitro*–*derived ULF following tandem mass tag (TMT)-mass spectrometry analysis of the proteomic content following exposure to: vehicle control (green circle), 1 ng/mL Insulin (yellow circle), or 10 ng/mL Insulin (red circle) for 72 hours (n = 3 biological replicates). (B) Venn diagram analysis of overlap and unique proteins that are differentially abundant following exposure to different concentrations of insulin. Fold change differences in protein abundances between groups was determined using paired t-tests and were considered significant when *P* < .05.

**Figure 8.**
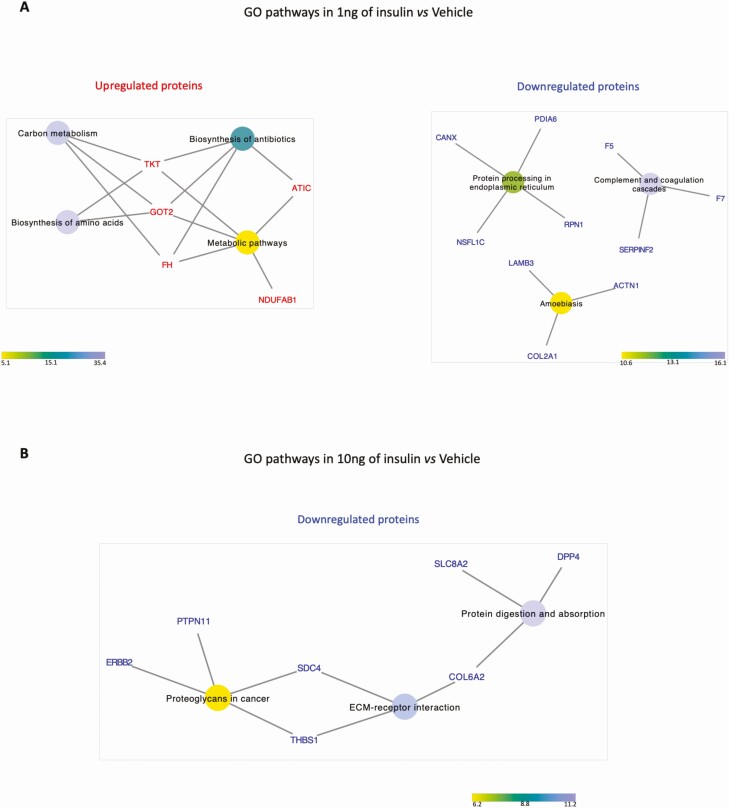
Network interaction between the differentially abundant proteins and their signaling pathways (*P* < .05) from in vitro*–*derived ULF from endometrium on-a-chip exposed to insulin for 72 hours. (A) GO pathways associated with proteins altered in abundance following treatment with 1.0 ng/mL of insulin compared to vehicle control. (B) GO pathways associated with proteins altered in abundance following treatment with 10.0 ng/mL of insulin was compared to vehicle control. The color circle represents an individual signaling pathway and the proteins around it are proteins identified as altered in our treatments associated with signaling pathway(s) that are either upregulated (red) or downregulated (blue) following insulin treatment. The color scale represents the fold enrichment that reflects the involvement of the proteins in each signaling pathway.

PCA did not reveal distinct clustering in the overall proteomic profile of in vitro*–*derived ULF (Fig. 7A). However, exposure of our endometrium on-a-chip to physiological extremes of insulin (1 and 10 ng/mL) changed the abundance of 195 proteins (Fig. 7B). The majority of these proteins (n = 67) were altered (*P* < .05) in cells treated with the 1 ng/mL of insulin compared with vehicle control (Fig. 7). The higher concentration of insulin altered 57 proteins in total (*P* < .05), while comparison of the 2 physiological extremes of insulin (1 vs 10 ng/mL) revealed 51 differentially abundant proteins (*P* < .05) proteins in in vitro*–*derived ULF. Venn diagram analysis showed that 17 proteins were altered in more than 1 group (Fig. 7B). Proteins upregulated following treatment with 1 ng/mL of insulin were overrepresented in pathways associated with biosynthesis of amino acids (n = 2), carbon metabolism (n = 3), biosynthesis of antibiotics (n = 4), and metabolic pathways (n = 5). The downregulated proteins were associated with complement and coagulation cascades (n = 3), protein processing in endoplasmic reticulum (n = 5), and amoebiasis (n = 3). Downregulated proteins were overrepresented when cells were treated with 10 ng/mL of insulin were related with protein digestion and absorption (n = 3), extracellular matrix (ECM)–receptor interaction (n = 3), and proteoglycans in cancer (n = 4). All the pathways related with up- or downregulated proteins are presented in Fig. 8.

## Discussion

This study aimed to generate a novel in vitro model to study the interactions between the maternal metabolic environment and endometrial function. Using a microfluidics approach to mimic the bovine endometrium in vitro, we identified the transcriptomic pathways that are changed by different physiologically relevant concentrations of glucose and insulin as well as the consequences for the secreted proteomic composition of the uterine luminal fluid produced in vitro. These provide evidence for the potential mechanism of action of 2 key manifestations of metabolic stress in circulation (glucose and insulin) independently on uterine function and allow improved understanding of how maternal nutritional stressors contribute to uterine dysfunction and ultimately early pregnancy loss.

This device is composed of predominantly pure population of epithelial cells along with a stromally enriched population allowing interaction between these 2 cell types to better mimic in vitro what occurs in vivo. Moreover, these cells express both steroid hormone receptors which represent the expression patterns observed in vivo in the early luteal phase of the estrous cycle (38). Concentrations of glucose and insulin are decreased in the circulation of dairy cows experiencing negative energy balance (39, 40). In vivo experiments have investigated how the oviduct and endometrium as well as their secretions are modified by the metabolic status of the maternal environment (8, 9, 15, 17). While reduced blood glucose and insulin concentrations alone are not detrimental for fertility and embryo survival (41), they result in a modified uterine environment that has consequences for offspring health (15, 42). Lactation results in a complex metabolic environment which not only has low concentrations of glucose and insulin but elevated concentrations of nonesterified fatty acids and beta-hydroxybutyrate. Up to now, it has not been possible to determine the specific role played by nutritional stressors in modifying uterine function. Most in vitro models of bovine endometrium use traditional static cell culture or explants (25, 43-46) and while microfluidics have been used to recapitulate the human menstrual cycle (24) or the bovine oviduct (23), to our knowledge this is the first paper to report the use of microfluidics to mimic the bovine endometrium. This allowed us to determine the mechanism by which individual nutritional stressors alter endometrial function.

In early development, mammalian embryos use glucose as the main energy source to synthesize glycogen, nucleic acids, proteins, and lipids (47, 48). It is critical, therefore, that sufficient glucose is transported into the uterine lumen to support embryo development; however, glucose can also act to modify the transcriptional response of a cell which modifies the uterine environment. We demonstrated that exposure to altered concentrations of glucose changed the expression of transcripts involved in the biological processes of collagen fibril organization, blood vessel development, regulation of interferon α and β production, positive regulation of MAPK and ERK1/2 and positive regulation of energy homeostasis as well as the molecular functions of ECM structural constituent, platelet-derived growth factor binging. Platelet activation is a complex signaling pathway positively dependent on several components as well as glycoprotein Ib-IX-V complex (GPIb-IX), phosphoinositide 3-kinase (PI3K-Akt), immunoreceptor tyrosine-based activation motif (ITAM), mitogen-activated protein kinase (MAPK), extracellular signal-regulated kinases 1 and 2 (ERK1/2), among others (49). Interactions between cells such as epithelial and stromal cells of the endometrium, involve mechanisms and ECM constituents, including cell surface receptors (integrins) and receptors for fibronectin, collagen, and laminin. Stimulation of suspended platelets is an event dependent of collagen and thrombin and increase in intracellular Ca^2+^ is a key element in this process. Some of these events were connected with the GO terms involved with the DEGs when the endometrial cells were exposed to physiological extremes of glucose. Exposure to different concentrations of glucose not only altered DEGs in endometrial cells, but also altered the protein composition of in vitro ULF. This included proteins involved in platelet activation and lysosome pathways.

Platelet activation plays a critical role in the function of platelets and it is involved in coagulation and inflammatory processes. Normally, platelet activation is induced by collagen or soluble platelet agonists that bind to G protein receptors, which stimulates the activation of platelet receptors (integrin α _IIb_β _3_), mediating platelet adhesion and aggregation (49). Interestingly, the platelet-activating factor, a potent lipid mediator of inflammation and allergy, is involved in several reproductive processes (50) and platelet-activating factor receptors are present in the oviduct of hamsters (51) and mice (52) and the oviduct and endometrium of cows (53). In humans, PFA increases vascular permeability and vasodilation, necessary processes for embryo implantation and plays an important paracrine role in stromal and epithelial cells interactions during this process (50). We have shown components of this pathway are altered by glucose and propose that may contribute to reduced endometrial function associated with altered glucose concentrations.

The other signaling pathway related with the proteins from the in vitro ULF is the Lysosome pathway. The lysosome pathway is the primary site of cell digestion, lysosomes support cell function, recycling and providing a set of metabolites, such as amino acids, saccharides, lipids, ions, and nucleobases and a key integrator and organizer of cellular adaptation and survival (54). Lysosomes link important metabolic processes encoded by AMPK (adenosine monophosphate-activated protein kinase) and GSK3β (glycogen synthase kinase 3) signaling hubs. AMPK is a primary cellular sensor for energy stress and glucose levels, promoting catabolic programs in response to low energy levels (55). AMPK has been linked to endometrial cancer in humans and depending on the context, AMPK can promote proliferation or cellular death (56). In addition, GSK3β is a kinase with apparently contradictory functions (57); for example, the presence of GSK3β in lysosomes can stimulate cell growth and survival, while its presence in the nucleus can promote cell death functions (58). In humans, GSK3β is expressed in endometrial cells and GSK-3β phosphorylated form exhibits cyclic variation. Furthermore, phosphorylation of GSK-3β is regulated by progesterone and the inhibition of GSK-3β is temporally regulated with the increased glycogen synthesis in the endometrial cells during the luteal phase (48, 59). Thus, incorrect inactivation of GSK-3β could result in inadequate glycogen production and could potentially affect embryo implantation (48). In a previous study with dairy cows, increasing the circulating energy substrate, by exogenous infusion of glucose, was directly associated with a decrease in embryo development (size, width, and area) (42), in contrast to what was expected. We propose that physiological glucose extremes may directly affect these important signaling pathways (platelet activation and lysosome) and interfering in processes such as receptivity and embryonic implantation.

Insulin plays an essential metabolic role in regulating energy homeostasis in the body and insulin-dependent signaling also has key functions in reproductive events and early development. In cattle, insulin concentrations vary throughout the estrous cycle (60); however, when an embryo is present in uterus, there is a decline insulin concentrations in ULF (61). The bovine pre- and peri-implantation embryo expresses receptors for IGF and insulin receptors (62, 63) indicating the embryo is sensitive to concentrations of insulin during pre-implantation development (61). Depending on the nutritional status of the cow, insulin is suggested to have a metabolic function, regulating glucose levels in the uterus (64). Microfluidic exposure of endometrial cells to different concentrations of insulin in vitro in this study altered signaling pathways in both epithelial and stromal cells related to metabolism. In vivo, low circulating levels of insulin are associated with negative energetic balance and the consequence of these low concentrations range from a delay in ovulation to an unfavorable environment for embryo development and even for the pregnancy maintenance (61). On the other hand, high concentrations of insulin, although favorable for ovulation, are detrimental for early embryo development (65, 66). Insulin supplementation during in vitro development has led to conflicting results with some improvement to morula stage embryos reported and some increase in cell number in the blastocysts, but most authors did not observe any effects on the blastocyst rate—a key developmental checkpoint (67-72). Even in the absence of effects on blastocyst development, an improvement in the number of cells in embryos could indicate a beneficial effect on the establishment of pregnancy. This may occur since blastocysts are able to produce and release embryotropins involved in modulating endometrial transcripts mediated by interferon-tau and prostaglandin metabolism (73, 74). However, to the best of our knowledge, the effects of insulin during the early luteal phase in in vitro bovine endometrial cells had not previously been investigated.

We observed that components of the complement and coagulation cascade were altered when the endometrium on-a-chip was exposed to a low concentration of insulin. The complement system is involved in the innate immune system and mostly functions to remove pathogens, dead cells, and debris. During early pregnancy there are changes to immune cells and markers in the endometrium (75, 76). In ruminants, receptivity to implantation involves a set of orchestrated events, among which is the suppression of genes for immune recognition of the conceptus (embryo/fetus and associated membranes) and the increase in vascularization of the endometrium (reviewed by (77)). Exposure to different concentrations of insulin may modify these processes and contribute to dysregulated endometrial function.

Exposure of endometrial cells to physiological extremes of insulin also changed the composition of proteins in the in vitro*–*derived ULF. In general, the proteins up-regulated in low insulin concentration were associated with signaling pathways mainly related to metabolic events (carbon metabolism, biosynthesis of amino acids, biosynthesis of antibiotics and metabolic pathways). The proteins downregulated when the endometrial cells were exposed to high concentrations of insulin were associated with protein digestion and absorption, ECM–receptor interaction, and proteoglycans in cancer. Proteoglycans (including syndecan-4 which we observed in our study) are often found on the cell surface or in the ECM and perform multiple functions in cancer and angiogenesis through their ability to interact with both ligands and receptors that regulate neoplastic growth and neovascularization (78). Syndecan-4 connects 2 important signaling pathways (proteoglycans in cancer and ECM–receptors interactions) and it has been reported that blocking interactions between syndecan-4 and fibronectin decreases focal adhesions in cells, leading to increased cell proliferation in tumors. Thus, syndecan-4 has a key role in regulating cell adhesion, migration and proliferation in some tumors (79). The protein thrombospondin-1 (THBS1) was also associated with the ECM-receptor interaction pathway. This glycoprotein can bind to fibrinogen, fibronectin, laminin, collagen types V and VII, and integrins mediating the interactions between cells and ECM. Also, THBS1 is involved in regulation of angiogenesis (80).

In humans, changes in ECM and/or in ECM-related signaling pathways are often attributed to pathological events, including premature birth, cervical incompetence, endometriosis, polycystic ovary syndrome, and neoplasms in the reproductive tract (81). Thus, demonstrating that ECM–receptor interactions pathway can be also involved in other tissues and biological events. In ruminant livestock species, it was previously reported that the characteristic posthatching elongation of the conceptus requires interaction between the trophectoderm and the uterine luminal epithelium that causes a mosaic of interactions between integrins and ECM, which act together to promote adhesion during implantation (82, 83). While our investigations were carried out in the pre-elongation stage of development, insulin-regulated changes to ECM components of the endometrium in early development may contribute to a compromised uterine environment and pregnancy loss. We found limited correlation between the transcriptomics signature of the epithelial cells and the protein content of the secretome. This is not unusual given differences in rates of translation from mRNA into proteins as well as protein turnover and degradation rates (84). Moreover, there is an order of magnitude in the difference of detection of transcripts via RNAseq and proteins via mass spectrometry likely contributing to the differences observed.

In summary, we are the first to report the use of microfluidics to mimic the bovine endometrium in vitro. We demonstrate that this approach allows us to determine how alterations in individual components of the maternal metabolism impact endometrial function. We specifically demonstrate at the transcriptional and proteomic levels that altered concentrations of glucose and insulin change ECM components of the endometrium. We propose that changes to these ECM components contribute to the compromised uterine environment associated with metabolic extremes in maternal circulation.

## Data Availability

The mass spectrometry proteomics data have been deposited to the ProteomeXchange Consortium via the PRIDE partner repository with the dataset identifier PXD024218 (https://www.ebi.ac.uk/pride/archive?keyword=PXD024218). All RNA sequencing data are available via GEO database number GSE167086 (https://www.ncbi.nlm.nih.gov/geo/query/acc.cgi?acc=GSE167086).

## Abbreviations

AGC: automatic gain control
AMPK: adenosine monophosphate-activated protein kinase
ECM: extracellular matrix
FDR: false discovery rate
FTMS: Fourier transform mass analyzer
GO: gene ontology
Ig: immunoglobulin
IGF: insulin-like growth factor
LC: liquid chromatography
MS/MS: tandem mass spectrometry
PBS: phosphate-buffered saline
PCA: principal component analysis
RT: room temperature
TMT: tandem mass tagging
ULF: uterine luminal fluid

## Additional Information

***Disclosures:*** The authors have nothing to disclose.
